# Osteogenic Differentiation Effect of BMP-9 with Phenamil and Simvastatin on Intact Human Amniotic Epithelial Stem Cells

**DOI:** 10.52547/ibj.3748

**Published:** 2022-10-29

**Authors:** Armin Ahmadi, Seyed Shayan Ebadi, Tahereh Tayebi, Seyed Alireza Ebadi, Mohammad Mahdi Sarzaeem, Hassan Niknejad

**Affiliations:** 1 Department of Pharmacology, School of Medicine, Shahid Beheshti University of Medical Sciences, Tehran, Iran;; 2 Department of Internal Medicine, School of Medicine, Shahid Beheshti University of Medical Sciences, Tehran, Iran;; 3 Department of Orthopedic Surgery and Traumatology, Imam Hossein Medical Center, Shahid Beheshti University of Medical Sciences, Tehran, Iran; † Co-first author

**Keywords:** Amniotic Membrane, Phenamil, Simvastatin, Tissue engineering

## Abstract

**Background:**

Bone tissue engineering has shown to be a promising strategy for repairing bone defects without causing harmful side effects to the patient. Three main building blocks of tissue engineering, including seeding cells, scaffold, and signaling molecules, are required for adequate bone regeneration. The hAM is the innermost of the placental membranes. In addition to providing a source of stem cells and growth factors, hAM has several features that make it an appropriate scaffold containing stem cells for use in tissue engineering purposes. The present investigation aimed to assess the effect of BMP-9 combined with phenamil and simvastatin on osteogenic induction of hAM with its hAECs.

**Methods:**

Using six different OMs, we cultured hAM for 14 days. The basic OMs were chosen as the first group and other media were made by adding BMP-9, phenamil, simvastatin, BMP-9 alongside phenamil, and BMP-9 alongside simvastatin to the basic OMs. Finally, viability assay, tissue mineralization, calcium and phosphate content determination, and measurement of LDH, and ALP activity were performed.

**Results:**

Among all study groups, groups containing simvastatin showed a significantly lower level of viability. Although all media could induce osteogenic features, the hAECs cultured in media containing BMP-9 and phenamil demonstrated a wider area of mineralization and a significantly higher level of calcium and phosphate content, LDH, and ALP activity.

**Conclusion:**

Our findings indicated that the use of phenamil together with BMP-9 could synergistically show *in situ* osteogenic induction in hAECs, which could be a new insight into translational medicine.

## INTRODUCTION

Reconstruction of bone defects resulting from traumas and therapeutic resections is still a major challenge during orthopedic and maxillofacial surgeries. Bone regeneration with bone grafts, including autografts, allografts, and xenografts, is limited by various issues, such as donor site morbidity, lack of revascularization, and bone graft rejection. To overcome these difficulties, the development of bone tissue engineering utilizing optimal scaffolds seeded with stem cells has emerged as a promising strategy^[^^1^^]^. Based on the principles of bone tissue engineering, seeding cells, scaffolds, and signaling molecules are three main building blocks for the bone contributing to its renewal^[^^2^^]^. Hence, in order to achieve optimal results in the clinic, novel options for bone engineering need to be explored. 

Various types of growth factors, including BMP 2, 4, 6, 7, and 9, insulin-like growth factor 1, vascular endothelial growth factor, and fibroblast growth factor, are available for the differentiation of stem cells into mature bone cells^[^^3^^]^. In bone tissue engineering, BMPs have been unveiled as one of the most widely used and crucial factors in the regulation of bone homeostasis and osteogenic differentiation^[^^4^^]^. Among BMPs, BMP-9 has been demonstrated to be the most potent osteoinductive BMP both *in vivo* and *in vitro*^[^^5^^-^^7^^]^. Hence, utilization of BMP-9 alongside other BMPs could have synergistic effects on cell differentiation. However, since the procedure of growth factor production is an expensive one, utilization of recombinant growth factors has been faced with some limitations. In the last decade, small molecules have been considered as suitable alternatives to growth factors in tissue engineering^[^^8^^,^^9^^]^. Among the small molecules, phenamil and statins have osteoinductive effects during cell differentiation^[^^10^^,^^11^^]^. Phenamil, an FDA-approved derivative of amiloride, a potassium-sparing diuretic drug, is an activator of BMP signaling pathway. Phenamil exerts its pro-BMP effects via the activation of tribbles homolog 3 and subsequent stabilization of SMAD, a main transcription factor in BMP signaling^[^^12^^]^. Statins are among the most widely used and highly available components with negligible side effects^[^^13^^]^. It has previously been found that the use of statins in OM could improve the differentiation of stem cells into osteoblast^[^^11^^,^^14^^,^^15^^]^. Lipophilic statins, particularly simvastatin, have been considered the best choice for osteogenic differentiation^[^^16^^]^.

HAM is the innermost layer of the placental tissue^[^^17^^]^ and comprises two types of stem cells: amniotic epithelial cells and amniotic mesenchymal stromal cells^[^^18^^]^. It also contains growth factors and possesses anti-inflammatory and anti-bacterial characteristics, making hAM a proper candidate for tissue engineering studies. Previously, hAM and its products have been investigated as a method of treatment for several diseases. Application of hAM in the treatment of refractory plantar fasciitis has shown considerable effects comparable to corticosteroid injection. HAM has also been used in the treatment of knee osteoarthritis and as a wrap in tendon defects, enhancing tendon repair after damage^[^^19^^]^. Likewise, hAM-derived products have been used to treat critical-sized bone defects in an animal model^[^^20^^,^^21^^]^. Several studies have indicated that both intact^[^^22^^]^ and isolated^[^^23^^]^ hAECs possess the potential to be differentiated toward osteogenic lineage^[^^24^^]^. The hAM possesses mechanical features appropriate for utilization as a scaffold. As the hAECs are spread naturally on the AM surface, application of BMP-9, simvastatin, and phenamil in hAM, as an innate source of stem cells spreading on their own basement membrane as a natural scaffold, could be an effective method in bone tissue engineering. Therefore, the aim of this investigation was to evaluate the osteoinductive effect of BMP-9 with phenamil and simvastatin on intact human amniotic epithelial cells in order to create an osteoblast containing scaffold for use in the management of bone defects due to trauma such as nonunion bone fractures. To our knowledge, this is the first study to investigate the effects of the combination of BMP-9 and either of the two small molecules simvastatin and phenamil on amniotic epithelial cells differentiation into osteogenic lineage.

## MATERIALS AND METHODS

**Preparation and cultivation of hAM**

**Fig. 1:**
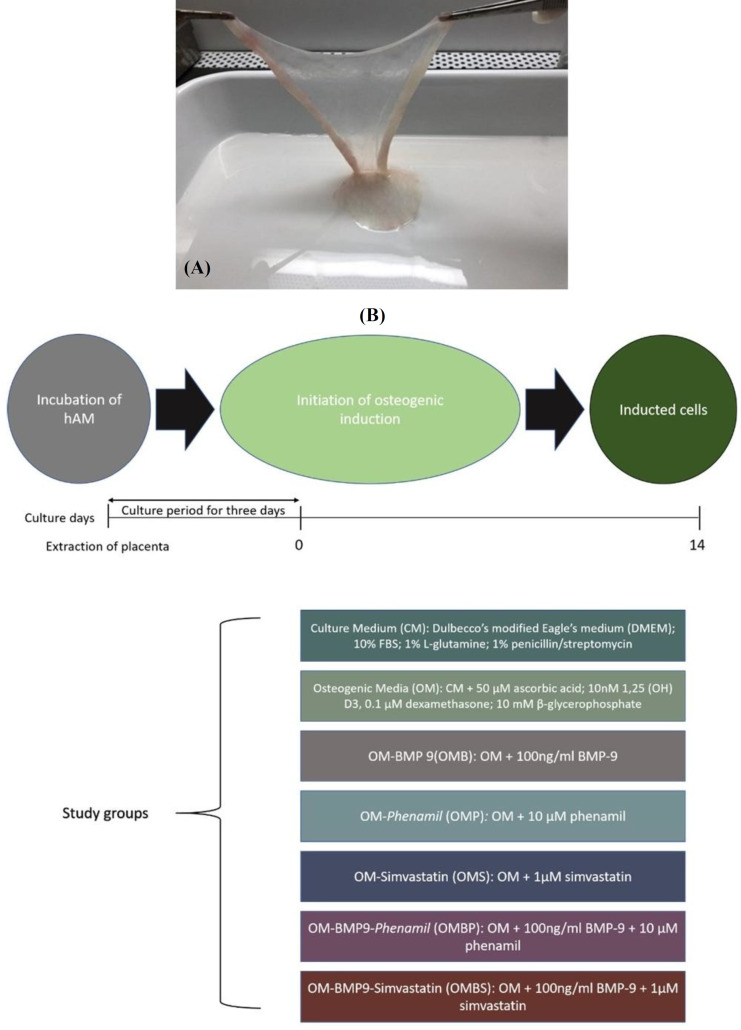
Intact hAM was peeled manually by blunt dissection (A) osteogenic induction procedure and the study groups in detail (B)

 The placenta was collected through elective cesarean sections from healthy mothers. The mothers who showed no pathologic features including diabetes mellitus, membrane premature rupture, preeclampsia, urogenital infections, preterm or postterm cesarean, placental abruption, placenta previa, or placenta accreta, were excluded from the study. The placentas were kept in Ringer’s lactate serum blended with antibiotic solution (1% penicillin/streptomycin, Thermo Fisher, USA) inside a sterile plate at 4 °C to be further processed. Then the hAM was peeled off manually from placenta by blunt dissection and washed thoroughly with cold PBS (Fig. 1A). Subsequently, hAM was dissected with eight-mm punches into equal pieces and cultured for three days using DMEM (Gibco, UK), 10% FBS (Sigma-Aldrich, USA), 1% L-glutamine (Sigma-Aldrich), 1% penicillin/streptomycin (Thermo Fisher) in 12-well plates with 95% air and 5% CO_2_ at 37 °C.

**Osteogenic induction**

All the hAM pieces were cultured for three days in CM. For osteogenic induction, hAM pieces were divided into six groups and treated with treatment media containing OM, DMEM, 10% FBS, 1% L-glutamine, and 1% penicillin/streptomycin, as well as 50 µM of ascorbic acid, 10 nM of 1,25(OH)D_3_, 0.1 µM of dexamethasone, 10 mM of β-glycerophosphate, OMB (OM + 100 ng/ml of BMP-9), OMP (OM + 10 µM of phenamil [Sigma-Aldrich]), OMBP (OM + 100 ng/ml BMP-9; 10 µM of phenamil), OMS (OM + 1 µM simvastatin), and OMBS (OM + 100 ng/ml of BMP-9; 1 µM of simvastatin). Moreover, a control group was considered and kept in CM under the same condition as the other groups. The media were changed three times weekly. The tissues were treated for 14 days using Oms, and the outcomes were measured at the baseline and after treatment. Osteogenic induction procedure is illustrated in Figure 1B.

**Cell viability assay**

 MTT (Sigma-Aldrich) was used to evaluate the effect of osteogenic induction on the viability of hAECs at day 14 of osteogenic induction. The punched biopsies were incubated in the media in which they were previously cultured containing 5 mg/ml of MTT solution for 4 h. Subsequently, the formazan crystals, formed by mitochondrial dehydrogenases of living cells, were dissolved by the addition of dimethyl sulfoxide (Merck, Germany). The absorbance of the dissolved formazan crystals was measured at 570 nm using an ELISA reader. Finally, the percentage of viability of fresh hAECs was calculated at day 0. 

**Histology and mineralization of biopsies**

 After 14 days of osteogenic induction, the cultured cells were fixed by adding 4% paraformaldehyde for 10 minutes. Then the cells were rinsed completely with PBS solution, and the biopsies were obtained from all treatment groups at day three and after osteogenic induction. Subsequently, mineralization of the mentioned biopsies was evaluated using AZ staining. staining of the biopsies was observed under a light microscope. The stained area was measured using Image J software, and the percentages of all areas of punched biopsy were calculated.

**Calcium and phosphate content of hAECs**

 To measure the calcium content of hAECs following osteogenic induction, the punched biopsies were transferred to the wells containing 200 µl of 0.5 M hydrochloric acid (Sigma-Aldrich) and shaken at 488 rpm at 37 °C. Following three hours of shaking, the suspension was centrifuged at room temperature at 180 ×g for 12 minutes. Then the calcium content of hAECs was measured by photometric method at 570 nm using Calcium CPC FS Kit (DiaSys Diagnostic Systems, Germany). With the aim of evaluating phosphate content of hAECs, hAM biopsies were washed completely by cold TBS (Sigma-Aldrich) and resuspended in 1 mL of TBS. The prepared tissues were subsequently sonicated using an ultrasonic homogenizer for 5 minutes. Afterwards, the suspensions were centrifuged at room temperature for 12 minutes and the phosphate content of hAECs was evaluated using Phosphate Assay Kit (Abcam, Cambridge, UK) by colorimetric method. 

**ALP activity assay**

To evaluate the osteogenic effects of the treatment groups, ALP activity was measured at the days 0 and after day 14 of osteogenic induction. The cultured punched biopsies were washed with cold TBS and lysed using 0.25% Triton-X100 (Sigma-Aldrich) for two minutes with three repeats. Subsequently, p-Nitrophenylphosphate (Sigma-Aldrich) was added to the punched biopsies and incubated for 60 minutes. Next, the absorbance was measured using ELISA reader (BioTek, USA) at 405 nm. The ALP activity of hAECs was calculated using the standard curve of p-NP (Sigma-Aldrich). In order to distinguish between placental ALP (heat stable) and bone ALP (heat labile)^[^^25^^]^, the biopsies from each group were warmed at 56 °C for 10 minutes, and ALP activity was again measured. 

**LDH assay**

 The LDH assay was performed to measure the intracellular LDH in hAECs at the days 0 and 14. LDH was recognized as a marker of progression in osteogenic differentiation^[^^26^^]^. The incubated hAM tissues were washed thoroughly with cold PBS and suspended in cold LDH assay buffer (Abcam). Afterward, the AM tissues were homogenized using an ultrasonic homogenizer for 5 minutes and centrifuged at 180 ×g at 4 °C for 15 minutes. The intracellular LDH was measured by colorimetric method using LDH assay kit (Abcam). 

**Statistical analysis**

 The statistical analysis was performed using SPSS version 26. The results were provided as mean ± SD, and analysis of variances (one-way ANOVA) with Tukey’s test was used to determine the statistically significant difference between the groups. *p* value less than 0.05 was considered as statistically significant.

**Fig. 2:**
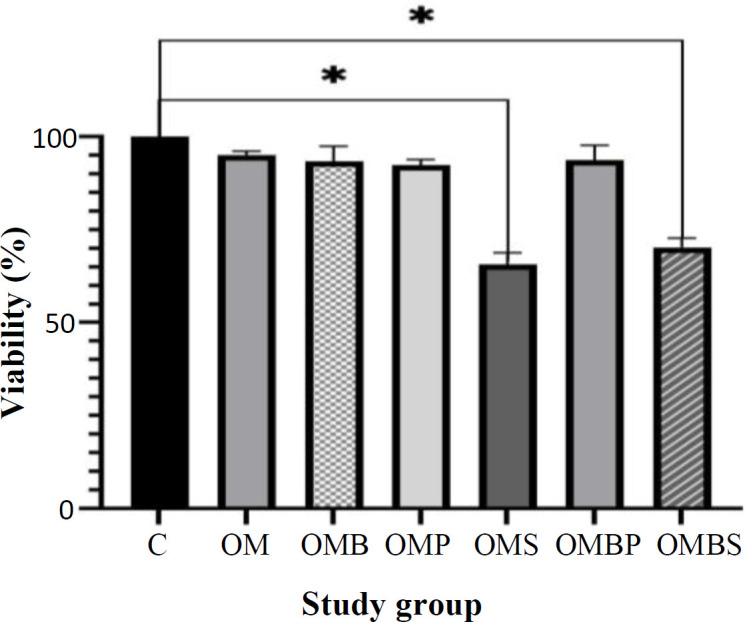
The viability assay of hAECs following 14 days of osteogenic induction. The viability was evaluated using MTT and compared with fresh amniotic membrane at day 0. hAECs in simvastatin-containing groups including OMS and OMBS had lower level of viability compared to control group. There is no significant difference between the control and other groups (^*^p < 0.05). C, control

## RESULTS

**Viability of hAECs after osteogenic induction**

 Viability assay was performed using MTT to evaluate the effect of each medium on the viability of hAECs. Following osteogenic induction, no significant difference was observed in terms of the viability of hAECs in the OM, OMB, OMP, and OMBP groups compared to the control. However, the mean ± SD of the viability of hAECs in simvastatin-containing groups, i.e., OMS and OMBS, was significantly lower than the control group (*p *< 0.05). At the end of the study, the viability of hAECs in all study groups is shown in details in Figure 2.

**Histological analysis of the hAECs by AZ**

**Fig. 3:**
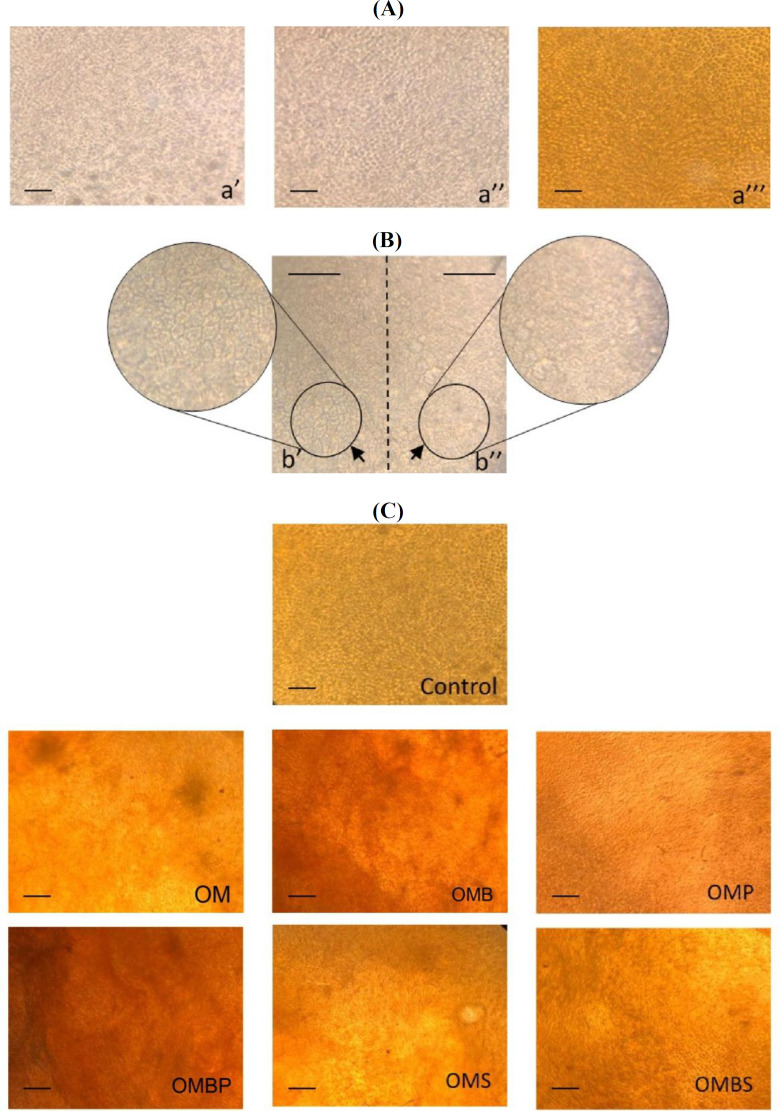
hAECs before osteogenic induction (A): at the day 0 (a’), at the day three after cultivation in CM (a’’), at the day three after cultivation in CM and staining by AZ (a’’’). The hAECs appearance after osteogenic induction (B); the cells that were in the richly stained side of the AM had polygonal appearance and seeded regularly (b’), whereas poorly stained side had amorphic and irregular cells (b’’). hAECs after osteogenic induction and staining by AZ (C); osteogenic media (OM), OM with BMP-9 (OMB), OM with phenamil (OMP), OM with simvastatin (OMS), OM with BMP-9 and phenamil (OMBP), OM with BMP-9 and simvastatin (OMBS). Scale bars = 50 µm

 To determine the morphology of hAEC, the hAM pieces were observed using light microscopy prior to culturing at day 0 and after day 14. After three days of cultivation with CM, no significant difference was observed in the morphology of hAEC. Moreover, three days after initiation of the experiment, no deposition of mineralization was detected using AZ staining (Fig. 3A). Then the groups were treated by the treatment media for 14 days. Following osteogenic induction, the mineralization of the treated tissues was not homogenously distributed; however, the mineralization pattern was found to be linear or patchy. The hAECs, which were located on richly colored areas, showed polygonal and more regular appearance compared to the other cells (Fig. 3B). In order to compare the distribution of mineralization among the study groups, the Image J software was used. Tissue mineralization was observed in all study groups, except for the control group. Moreover, all the BMP-9-containing groups, including OMB (36.9% ± 2.3%), OMBP (66.2% ± 2.9%), and OMBS (36.0% ± 1.7%), showed a wider area of mineralization compared to OM (23.1% ± 3.1%), with *p* < 0.001. OMBP group indicated a significant wider area of mineralization compared to all study groups (*p* < 0.001); however, mineralization of OMS and OMP groups was not higher than the OM group (Fig. 3C).

**Biochemical markers**

**Fig. 4:**
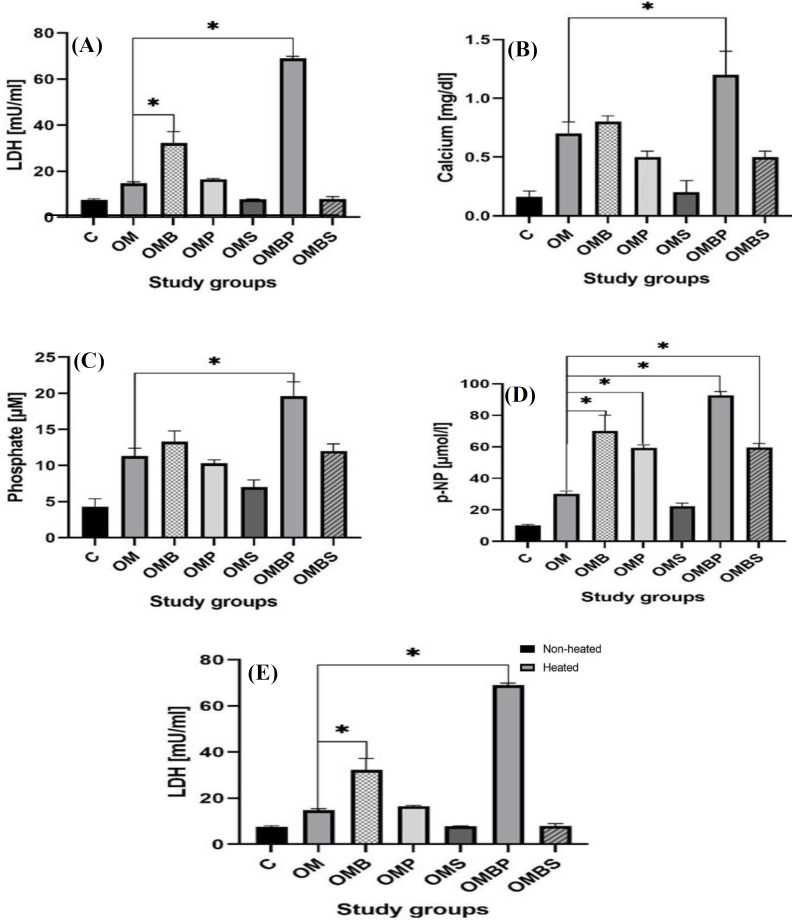
Biochemical markers of the treatment and control groups demonstrated as mean ± SD. (A), LDH, (B) calcium content,  (C), phosphate, (D) ALP activity; (E) ALP activity level of treatment and control groups before and after heating at 56 °C for 10 minutes shown as mean ± SD (^*^p < 0.05)

 After 14 days of osteogenic induction, all the measured biochemical markers of control group, including calcium, phosphate, ALP and LDH activity did not change significantly compared to day 0 (Table 1). To determine the effect of OM on osteogenic induction of hAECs, the osteo-representing biochemical markers were measured and compared between the OM and control groups. Following 14 days of osteogenic induction, the mean ± SD of calcium level in the OM group significantly increased compared to the control group (0.7 ± 0.1 mg/dl versus 0.1 ± 0.05 mg/dl, respectively; *p* < 0.001). Moreover, a significant increase was observed in phosphate content, LDH, and ALP activity (p-NP) of OM group compared to the control group (11.3 ± 1.1 µM versus 4.3 ± 1.1 µM [*p* < 0.05], 14.8 ± 0.6 versus 7.5 ± 0.5 mU/ml [*p* < 0.05], and 30.0 ± 2.0 µmol/l versus 10.3 ± 0.5 µmol/l [*p* < 0.01], respectively). All the evaluated biochemical markers showed significant increase during osteogenic induction in the OMB, OMP and OMBP groups compared to the control group. However, groups containing simvastatin displayed different patterns following osteogenic induction. Neither OMBS nor OMS showed higher levels of LDH activity compared to the control group. Moreover, none of the osteo-representing biochemical markers increased in OMS group by osteogenic induction, but the levels of calcium, phosphate, and ALP activity increased in the OMBS group after 14 days of osteogenic induction (Table 1). In order to determine the effect of BMP-9, phenamil, and simvastatin on osteogenic induction, level of the osteo-representing biochemical markers of the study groups were compared with those of the OM. Following osteogenic induction, the ALP activity level of all the groups containing BMP-9 was significantly higher than that of the the basic OM group (*p* < 0.001). Furthermore, phenamil and BMP-9 exhibited similar levels of ALP activity, both of which were increased compared to OM. Overall, all of the study groups demonstrated a significant higher level of ALP activity compared to the OM group, except for OMS (Fig. 4A). After 14 days of osteogenic induction, the between-group analysis revealed that calcium content of the cells treated with OMBP medium was significantly higher than that of the cells treated with OM (1.1 ± 0.2 mg/dl versus 0.7 ± 0.1 mg/dl, respectively; *p* < 0.001). However, the calcium content of the tissues cultured in OMB, OMS, OMP, and OMBS was not higher than that of OM (Fig. 4B). Likewise, among all study groups, only the group containing BMP-9 alongside phenamil had a significant higher level of phosphate than that of OM (19.6 ± 2.0 µM versus 11.3 ± 1.1 µM, respectively; *p* < 0.001; Fig. 4C). Regarding LDH evaluation, OMB and OMBP groups showed a higher level of LDH activity compared to the OM group (*p* < 0.001). Furthermore, the groups containing BMP-9 alone and BMP-9 with phenamil (OMB and OMBP) had significantly lower level of LDH activity compared to OM group (*p* < 0.01; Fig. 4D). In order to determine the type of isozyme of ALP, including placental (heat-stable) and liver/bone/kidney (heat-unstable), the specimens were warmed, and the ALP activity was measured once again. After 10 minutes of heating, the ALP activity level in all study groups significantly decreased, except for the OMS group. The level of ALP activity in all study groups following heating is exhibited in figure 4B. The synergistic effect of phenamil on BMP-9 was evaluated by comparing the osteo-representing biochemical markers in the OMB group with those of the OMBP group. All evaluated biochemical markers, including calcium, phosphate, LDH, and ALP activity, were significantly higher in the group containing phenamil alongside BMP-9 than BMP-9 alone. The differences of the mentioned biochemical markers in all of the study groups are presented in Tables 2 and 3. 

**Table 1:** Biochemical markers of control group at days 0 and 14

**Variables**		**Control group**		***p*** **value**
		**Day 0**	**Day 14**	
CA (mg/dl)		0.2 ± 0.1	0.1 ± 0.05	0.98
PH (µM)		5.1 ± 0.5	4.3 ± 1.1	0.99
LDH (mU/ml)		8.0 ± 0.5	7.5 ± 0.5	0.96
p-NP (µmol/l)		11.2 ± 1.1	10.3 ± 0.5	0.97

CA, calcium; PH, phosphate; ANOVA, post Tukey's test, the data is represented as mean ±SD

## DISCUSSION

The present study for the first time revealed that our basic OMs could induce osteogenic features among sessile hAECs. Increase in the level of ALP activity, phosphate content, and LDH activity, as well as calcium content compared to control group implied that intact hAM could be used as a potential source of stem cell-scaffold complex. Previously, other types of stem cells including human bone marrow mesenchymal stem cells^[^^27^^]^, adipose derived stem cells^[^^28^^]^ and dental pulp stem cells^[^^29^^] ^have been differentiated into osteogenic lineages by using the same OM. These findings indicate that sessile hAECs have the potential for osteogenic differentiation similar to the other stem cells. Applying hAM as a scaffold for tissue engineering has largely been investigated in skin and wound healing^[^^30^^]^, but its role in bone tissue engineering has not comprehensively been discussed. An *in vivo* study suggested that implantation of acellular hAM in femoral bone defect in rats improved bone regeneration^[^^31^^]^. Moreover, application of amniotic membrane-derived biomaterials extensively repaired the femoral bone defects of rats compared to the commercial bone grafts alone^[^^32^^]^. These studies demonstrated that hAM could be effective in repairing animals bone defects. HAECs, which are naturally placed on the basement membrane of amniotic membrane, express embryonic stem cell specific markers, including SSEA-3 and SSEA-4, and specific transcription factors of pluripotent stem cells, including Oct-4 and Nanog^[^^33^^]^. In another study, it has been displayed that intact hAM could be used as a new material for differentiation of its sessile stem cells into osteogenic lineage^[^^22^^]^, which is completely in line with our study. Therefore, intact hAM could be considered as an easy-to-use, highly available and innovative source of scaffold-stem cell complex for bone tissue engineering. 

As another finding of the present study, application of BMP-9 could significantly induce higher level of osteogenic features compared to our basic media. A wider area of mineralization was observed in the groups cultured with BMP-9 compared to the other media used in this study. In addition, utilization of BMP-9 increased the level of osteo-representing biochemical markers, including LDH and ALP activity, compared to the basic media. To the best of our knowledge, there is no previous study on evaluating the effect of BMP-9 on sessile or non-sessile hAECs. Previously, it has been shown that application of BMP-9 could induce osteogenic differentiation on placenta mesenchymal stem cells^[34]^, as well as it has osteoinductive effect on the immortalized calvarial mesenchymal stem cells^[^^35^^]^, periodontal ligament stem cells^[^^36^^]^, and adiposederived stem cells^[^^37^^]^. Therefore, it is suggested that BMP-9 acts as an effective osteoinductive factor for osteogenesis of hAECs.

In the present study, we tried to demonstrate the efficiency of two well-known small molecules, i.e., phenamil and simvastatin, on the osteogenic induction of intact hAECs. Our study exhibited that samples treated with phenamil plus BMP-9 had a higher level of mineralization compared to BMP-9 alone. Moreover, utilization of phenamil and BMP-9 combination could lead to the production of more osteo-representing biochemical markers, including calcium, phosphate, LDH and ALP activity in hAECs compared to BMP-9. These findings suggest that combination of phenamil with BMP-9 could synergistically increase the osteogenic induction of intact hAM. However, combination of phenamil alone with basic media could not show any specific osteogenic feature. Consistent with our study, it has been previously found that delivery of phenamil could increase the osteogenic differentiation of adipose-derived stem cells induced by BMP-2^[^^38^^]^. Moreover, in an *in vivo* study, delivery of phenamil together with BMP-2 could coordinately improve bone repair in a rat model of critical-sized bone defect^[^^39^^]^. The osteogenic feature of phenamil was mediated by expression of tribbles homolog 3, a strong inhibitor of Smad ubiquitin regulatory factor 1^[^^12^^]^. Hence, phenamil could enhance osteo-inductive features of BMP-9 through SMAD signaling pathway. On the other hand, phenamil decreases expression of PPARγ, a key regulator of adipogenesis^[^^39^^]^. Utilization of BMPs could lead to the overexpression of PPARγ and subsequent adipogenesis. Application of phenamil could reduce the unwanted adipogenesis effects of BMPs and its undesirable effects, including cyst-like bone void formation^[^^40^^]^. Therefore, phenamil not only synergistically increased osteogenicity of BMP-9 but also effectively inhibited unwanted adipogenicity effect of BMPs by the downregulation of PPARγ.

**Table 2:** Biochemical characteristics of OMB and OMBP groups

**Variables**		**Groups**		***p*** **value**
		**OMB**	**OMBP**	
CA (mg/dl)		0.7 ± 0.1	1.2 ± 0.2	<0.01
PH (µM)		13.3 ± 1.5	19.6 ± 2.0	<0.01
LDH (mU/ml)		32.3 ± 4.9	69.0 ± 0.9	<0.001
p-NP (µmol/l)		70.0 ± 10.0	92.6 ± 2.5	<0.001

CA, calcium; ANOVA, post Tukey's test, the data is represented as mean ±SD; *p* value less than 0.05 is significant.

The small molecule simvastatin that we examined its osteogenicity showed no osteogenic features. Samples in the groups treated with simvastatin did not demonstrate a wider area of mineralization compared to the other groups. Moreover, calcium, phosphate, and LDH level of the cells treated with media containg simvastatin were less than that of the basic OMs. Our findings in respect to simvastatin-mediated osteogenesis are in contrary to previous findings revealing osteoinductive effect of simvastatin. Feng *et al.*^[^^41^^]^ and Zhang e*t al.*^[^^42^^]^ found that simvastatin could promote osteogenic differentiation in human mesenchymal stem cells. Furthermore, it has previously been explored that simvastatin has osteoinductive effect on human periodontal ligament stem cells^[^^43^^]^ and murine embryonic stem cells^[^^44^^]^. However, to the best of our knowledge, no study was identified to evaluate the osteoinductive effect of simvastatin on hAECs. A possible explanation for our findings regarding that simvastatin did not increase the osteogenic markers compared to OM, might be due to the difference of stem cells type compared to previous investigations. Moreover, lower level of LDH activity in the hAECs treated with the media containing simvastatin along with the lower viability of hAECs compared to the other groups suggest that the concentration of simvastatin used for osteoinduction in other stem cells could be cytotoxic for hAECs. In consistent with our results, Sabandal *et al.*^[^^45^^]^ explored that application of a similar concentration of simvastatin decreased the viability of human primary osteoblasts. Application of lower concentrations of simvastatin for osteoinduction of hAECs in the future studies would help reveal concentration-dependent effects of simvastatin on osteogenesis.

**Table 3:** Measurement of the biochemical markers after 14 days of osteogenic assay for all the study groups

**Groups**		**Biochemical markers**			
		**LDH ** **(mU/ml)**	**Ca** **(mg/dl)**	**PH** **(µM)**	**p-NP ** **(µmol/l)**
Control		6.5 ± 0.8	0.1 ± 0.1	4.1 ± 1.2	8.7 ± 1.7
Osteogenic media		13.7 ± 1.2	0.6 ± 0.1	11.2 ± 1.2	29.1 ± 2.3
Osteogenic media + BMP-9		31.3 ± 4.9	0.7 ± 0.1	13.3 ± 1.5	70 ± 10.0
Osteogenic media + phenamil		15.5 ± 0.6	0.4 ± 0.1	10.2 ± 0.6	58.8 ± 2.5
Osteogenic media + simvastatin		6.5 ± 0.3	0.1 ± 0.1	6.9 ± 1.1	21.2 ± 2.5
Osteogenic media + BMP-9 + phenamil		69 ± 0.9	1.2 ± 0.2	19.6 ± 2.0	92.6 ± 2.5
Osteogenic media + BMP-9 + simvastatin		6.51 ± 1.6	0.4 ± 0.0	12.0 ± 1.0	59.3 ± 2.6

CA, calcium; The data are represented as mean ± SD.

The present study disclosed that combination of the small molecule phenamil with BMP-9 could synergistically induce osteogenic features in hAECs. Parallel activation of osteogenic signaling pathways results in enhancement of differentiation. This synergistic effect is probably the main mechanism for the enhanced osteogenesis of phenamil and BMP-9. Despite activating similar signaling pathways, combination of simvastatin with BMP-9 failed to upregulate the osteogenic differentiation. Overall, intact hAM could be considered as a natural potential source of stem cell-scaffold complex for use in bone tissue engineering. Further studies would be required to evaluate these findings in animal models.

## DECLARATIONS

### Acknowledgments

The authors gratefully thank the Vice Chancellor for Research and Technology of Shahid Beheshti University of Medical Sciences, Tehran, Iran. The authors also would like to thank the personnel of Erfan Hospital (Tehran, Iran) for assistance in this research.

### Ethical statement

The study protocol was approved by the Ethics Committee of Shahid Beheshti University of Medical Sciences (SBMU), Tehran, Iran (ethical code: IR.SBMU.MSP.REC.1397.760). Written informed consents were obtained from all the parents who donated the placenta after elective cesarean. The identity and personal information of mentioned persons remained confidential.

### Data availability

Data supporting this article are included within the article.

### Author contributions

AA: conceptualization, performing cell culture, biochemical analysis and AR staining, writing the manuscript, revision; SSE: conceptualization, performing cell culture, biochemical analysis and AR staining, writing the manuscript; TT: Performing cell culture, biochemical analysis, supervision of lab work SAE and MMS: revision of the manuscript; HN: conceptualization, supervision of the the project, conceived the original idea and designed the study. All authors have read and approved the final version of the manuscript.

### Conflict of interest

None declared.

### Funding/support

Research reported in this publication was supported by Research Grant Committee from the National Institutes for Medical Research Development (NIMAD), Tehran, Iran [grant number: 4002480].
